# The response regulator CrsR positively regulates ansamitocin P-3 biosynthesis in *Actinosynnema pretiosum*

**DOI:** 10.3389/fmicb.2025.1684526

**Published:** 2025-11-04

**Authors:** Peipei Zhang, Gongli Zong, Tengfei Wang, Shuangying Zhao, Ruoyi Sun, Jiafang Fu, Meng Liu, Guangxiang Cao

**Affiliations:** ^1^Department of Epidemiology, The First Affiliated Hospital of Shandong First Medical University, Jinan, China; ^2^College of Biomedical Sciences, Key Lab for Genetic Engineering and Synthetic Biology of Shandong Province, Shandong First Medical University, Shandong Academy of Medical Sciences, Jinan, China; ^3^School of Municipal and Environmental Engineering, Shandong Jianzhu University, Jinan, China

**Keywords:** *Actinosynnema pretiosum*, ansamitocin, two-component system, biosynthesis, response regulator

## Abstract

Ansamitocin P-3 (AP-3), a maytansinoid antibiotic produced by *Actinosynnema pretiosum*, exhibits potent anticancer activity. However, its biosynthetic regulation in *A. pretiosum* remains largely unknown. Two-component systems (TCSs) are ubiquitous in *actinomycetes* and primarily regulate the biosynthesis of secondary metabolites. In this study, we identified a novel TCS, designated CrsRK, in *A. pretiosum* X47 through sequence analysis. Deletion of the response regulator gene *crsR* drastically decreased AP-3 production. RNA-seq revealed CrsR’s global regulatory role, significantly altering transcription of primary metabolic genes, especially those in purine metabolism. Crucially, the deletion of *crsR* also significantly downregulated transcription of the AP-3 biosynthetic genes, including *asm7*, *asm10–15*, *asm21*, *asm23–24*, *asmAB*, and *asm43–47*, which encode enzymes for multiple steps in AP-3 biosynthesis. Electrophoretic mobility shift assays confirmed direct binding of CrsR to promoters of *asm21*, *asm43–44*, and *asm45–47* operons, indicating direct transcriptional control. Our results demonstrate that CrsR positively regulates AP-3 biosynthesis by directly and indirectly controlling transcription within the AP-3 biosynthetic gene cluster. In conclusion, this study elucidates the critical role of CrsR in AP-3 biosynthesis and expands our understanding of AP-3 regulatory mechanisms and TCS functions in *A. pretiosum.*

## Introduction

Maytansinoid antibiotics are widely used as the cytotoxic “warhead” in antibody–drug conjugates (ADCs) due to their potent microtubule-depolymerizing activity (Lopus et al., 2010; Zafar et al., 2023). Ansamitocins were first isolated from *Nocardia* sp. C-15003 (now reclassified as *Actinosynnema pretiosum*), which has been established as its primary producing strain (Hasegawa et al., 1983; Higashide et al., 1977). Based on structural differences in the R substituent at the C-3 position, ansamitocins are classified into six derivatives: ansamitocin P-0, P-1, P-2, P-3′, P-3, and P-4. Among the ansamitocin derivatives, ansamitocin P-3 (AP-3) is the most abundant derivative in the fermentation yield and exhibits the highest biological activity, and its derivatives can be converted *in vitro* into the clinically used maytansinoid antibiotics DM1 and DM4 (Prota et al., 2014; Venghateri et al., 2013). These payloads are utilized in the FDA-approved targeted anti-tumor drugs, Kadcyla and Elahere, for treating metastatic breast cancer and platinum-resistant epithelial ovarian cancer, respectively (Modi et al., 2022; Narayan et al., 2021; Wedam et al., 2020). Therefore, microbial fermentation of AP-3 attracts considerable attention.

AP-3 biosynthesis is controlled by two genetic biosynthetic clusters (*asm* BGC) (Carroll et al., 2002; Yu et al., 2002). The pathway initiates with uridine diphosphate (UDP)-glucose, which is converted into 3-amino-5-hydroxybenzoic acid (AHBA) through the aminoshikimate pathway (Floss et al., 2011; Ghisalba and Nuesch, 1981; Kibby and Rickards, 1981; Yu et al., 2002). Increasing intracellular concentrations of UDP-glucose or the precursor methylmalonyl-ACP significantly enhances AP-3 production in *A. pretiosum* (Fan et al., 2016; Zhao et al., 2017). Subsequently, AHBA is condensed with three propionate units, three acetate units, and one glycosyl unit under the catalysis of type I polyketide synthases (PKSs) to form the proansamitocin. Following multiple post-modification steps, the proansamitocin is converted into AP-3 (Kubota et al., 2006; Li et al., 2011; Moss et al., 2002; Spiteller et al., 2003; Wu et al., 2011; Zhao et al., 2008). Furthermore, altering the fermentation medium or conditions significantly impacts AP-3 yield in *A. pretiosum* (Fan et al., 2014; Jia and Zhong, 2011; Lin et al., 2011).

Bacterial responses to environmental signals are generally regulated by sigma factors and two-component systems (TCSs). In TCSs, a membrane-localized kinase senses specific signals, autophosphorylates, and transfers the phosphate to a response regulator (Zschiedrich et al., 2016). The activation of the regulator influences target gene expression. TCS functions are well-studied in *Actinobacteria*, where multiple TCSs are involved in antibiotic biosynthesis. In *Streptomyces lincolnensis*, the TCS AflQ1/AflQ2 acts as a repressor of lincomycin biosynthesis, exerting its control through multiple downstream regulatory cascades (Wang et al., 2023). RspA1/RspA2 is directly involved in regulating the production of the polyether antibiotic salinomycin and primary metabolism in *Streptomyces albus* (Zhang et al., 2021; Zhang et al., 2020). MtrAB, a TCS in *actinomycetes*, plays a vital role in regulating antibiotic production. In *Streptomyces coelicolor*, deletion of MtrA resulted in a significant reduction in the biosynthesis of actinorhodin (ACT), undecylprodigiosin (RED), calcium-dependent antibiotic (CDA), and the yellow polyketide compound (yCPK) (Som et al., 2017b; Zhu et al., 2020). Similarly, in *Streptomyces venezuelae*, loss of MtrA function impaired the production of chloramphenicol (CHL) and jadomycin (JAD) (Som et al., 2017a). In *S. coelicolor*, single or double mutations in MacR/S largely inhibited ACT production while promoting aerial mycelium formation (Liu et al., 2021, 2019). In *Streptomyces gilvosporeus* F607, MacRS positively regulates natamycin biosynthesis and sporulation processes (Zong et al., 2022). DraR/K, another TCS in *S. coelicolor*, exhibits differential regulation, activating ACT biosynthesis while suppressing RED and yCPK production (Yu et al., 2012, 2014). Similarly, in *Streptomyces bingchenggensis*, AtcR/K functions as a global regulator coordinating multiple secondary metabolites (Yan et al., 2024). In the *A. pretiosum* X47 strain, TCS CNX_RS34865/CNX_RS34870 was found to regulate AP-3 biosynthesis, and the response regulator CNX_RS34870 positively modulates the expression of biosynthetic cluster genes and primary metabolic genes to enhance AP-3 production (Zhang K. et al., 2022). Furthermore, characterization of the PhoP/PhoR system in this strain reveals that PhoP acts as a negative regulator of morphological development, repressing the transcription of differentiation-associated genes, but does not affect AP-3 biosynthesis (Zhang P. et al., 2022).

In this study, we identified a novel TCS CrsR/CrsK in the genome of *A. pretiosum* X47 and generated a CrsR (response regulator) deletion mutant. Our findings demonstrate that CrsR deletion significantly impaired AP-3 biosynthesis. Furthermore, we demonstrated that CrsR directly activates the transcription of AP-3 biosynthetic genes (*asm43–44*, *asm45*, and *asm21*) to promote antibiotic production. These results elucidate CrsR-mediated regulation, providing a framework to improve AP-3 yields.

## Materials and methods

### Strains, plasmids, and culture conditions

For spore production, conjugation, and phenotype analysis, *A. pretiosum* X47 and derivatives were cultured at 30 °C on solid International Streptomyces Project-2 (ISP2) medium, Mannitol Soya Flour (MS) medium, and BSCA medium, respectively (Qin et al., 2017; Ma et al., 2007). The seed culture medium for *A. pretiosum* strains contained (w/v): glucose, 2%; soluble starch, 4%; soybean meal, 1%; polypeptone, 0.5%; NaCl, 0.3%; and CaCl₂, 0.5% (pH 7.0). The fermentation medium consisted of (w/v): maltodextrin, 3%; soluble starch, 3%; malt extract, 1%; polypeptone, 0.5%; and CaCl₂, 1% (pH 7.0). *Escherichia coli* DH5α (for cloning), BL21 (DE3) (for heterologous protein expression), and ET12567 (pUZ8002) (for conjugation) (Kieser et al., 2000) are cultured in Luria-Bertani (LB) or LB agar (LA) medium at 37 °C supplemented with appropriate antibiotics. The plasmids pET-15b (for *in vitro* expression of CrsR protein), pMD-18 T (subcloning vector for knockout or complementation plasmid construction), pJTU1278 (for gene knockout plasmid construction), pMS82 (for gene complementation plasmid construction), and pSET152 (template for the apramycin resistance cassette) (Bierman et al., 1992) were used in this study.

### *crsR* knockout in *Actinosynnema pretiosum* X47

To construct the *crsR* deletion mutant (ΔcrsR) in *A. pretiosum* X47, approximately 1.5 kb flanking regions upstream and downstream of the *crsR* gene were amplified from X47 genomic DNA using primer pairs crsR-L-F/L-R and crsR-R-F/R-R (Supplementary Table S1). The apramycin resistance cassette (*aac(3)IV*) was amplified from plasmid pSET152 using primers Apra-F/Apra-R (Bierman et al., 1992). The upstream flanking region, apramycin resistance cassette, and downstream flanking region were directionally assembled and ligated into pMD18-T using the ClonExpress II One Step Cloning Kit (Vazyme). The assembled fragment was subsequently excised from pMD18-T by digestion with *Xba*I and *Hind*III and cloned into pJTU1278, generating the deletion plasmid pM-crsR. For conjugal transfer, pM-crsR was introduced into *E. coli* ET12567(pUZ8002). This donor strain was then conjugated with *A. pretiosum* X47 as described (Kieser et al., 2000). Apramycin-resistant exconjugants were selected, and successful deletion of *crsR* was confirmed by polymerase chain reaction (PCR) using primers crsR-V-F/R.

### Genetic complementation of the *crsR* deletion mutant

For ΔcrsR complementation, a 2,344-bp fragment spanning the *crsRK* coding region and the native promoter of *crsK* was amplified from X47 genomic DNA using primers *crsR*-Com-F/R (Supplementary Table S1). The PCR product was cloned into pMD18-T and then subcloned as a *Hind*III fragment into pMS82 to generate plasmid pC-crsR. The resulting plasmid was transformed into *E. coli* ET12567 (pUZ8002), and the hygromycin B-resistant transformants were conjugated with the ΔcrsR strain. Exconjugants selected for hygromycin B were confirmed by PCR verification using primers V-F/R.

### HPLC quantification of AP-3 yield

The spore suspensions of *A. pretiosum* X47 and derivatives were inoculated into seed medium. Cultures were incubated at 28 °C with shaking at 220 rpm for 48 h. The seed cultures were transferred to fermentation medium and incubated at 28 °C and 220 rpm for 144 h. Culture supernatants of *A. pretiosum* strains were extracted with ethyl acetate. AP-3 was quantified by high-performance liquid chromatography (HPLC) using a Diamonsil C18 column (250 mm × 4.6 mm), an acetonitrile–water gradient mobile phase, and detection at 254 nm (Zhong et al., 2019). The dry cell weight (DCW) of *A. pretiosum* strains was determined by collecting 1 mL of culture supernatant, followed by centrifugation, removal of the supernatant, and drying the mycelia at 80 °C to a constant weight. The AP-3 production and DCW data for *A. pretiosum* strains were derived from three independent biological replicates, and the results are presented as the mean ± standard deviation.

### Total RNA isolation, RNA-Seq, and qRT-PCR assays

Spore suspensions of *A. pretiosum* strains were first cultivated in seed medium and then transferred to fermentation medium for an additional 48 h. Mycelia were harvested and processed simultaneously in a single batch. Total RNA was extracted from mycelia using TRIzol reagent (Invitrogen) according to the manufacturer’s protocol. RNA integrity and total RNA quantity were assessed using an Agilent 2,100 Bioanalyzer. Transcriptome sequencing was performed using NovoGene (Beijing, China) on the Illumina NovaSeq platform with 150 bp paired-end reads. Raw reads were processed using in-house Perl scripts to remove adapter sequences, reads containing N bases, and low-quality reads to obtain clean data. The quality of the clean data was evaluated by calculating Q20, Q30 scores, and GC content. All downstream analyses were performed using high-quality clean data. The reference genome was indexed using Bowtie2 v2.2.3, and the clean reads were aligned with the reference genome using the same tool. Differential gene expression between the X47 and the ΔcrsR mutant strains was analyzed using the DESeq2 R package. Genes with an adjusted *p*-value of <0.05 and |log₂(Fold Change)| ≥ 1 were considered significantly differentially expressed. Subsequent bioinformatic analysis followed the standard computational pipeline.

For qRT-PCR analysis, the X47 and ΔcrsR strains were cultured in fermentation medium for 48 h. Total RNA was then extracted using TRIzol reagent (Invitrogen) and reverse-transcribed into cDNA after genomic DNA removal using the PrimeScript FAST RT Reagent Kit with gDNA Eraser (RR092A, Takara). Amplification was performed on a LightCycler 480 instrument (Roche) using TB Green^®^ Premix Ex Taq™ (Tli RNaseH Plus) (RR420A, Takara) and gene-specific primers (Supplementary Table S1). The *hrdB* gene was used for normalization. Relative gene expression in the ΔcrsR mutant, presented as fold change compared to the X47 strain, was calculated from three independent biological replicates and is expressed as mean ± standard deviation.

### Recombinant protein expression and purification

The *crsR* gene was amplified and ligated into linearized pET-15b by homologous recombination using the ClonExpress II One Step Cloning Kit (Vazyme), yielding the His₆-tagged CrsR expression plasmid. This plasmid was transformed into *E. coli* BL21(DE3) competent cells. His-tagged CrsR expression was induced with 0.5 mM IPTG at 28 °C for 4 h. The protein was purified using Ni-NTA Sepharose 6FF resin (Sangon Biotech). Bacterial cells were resuspended in a lysis buffer supplemented with 20 mM imidazole and lysed via ultrasonication on ice. The clarified supernatant was applied to a Ni-NTA Sepharose 6FF column (Sangon Biotech). The column was initially washed with a buffer containing 100 mM imidazole to remove weakly bound and non-specific proteins. His-tagged CrsR was subsequently eluted in a buffer containing 250 mM imidazole. The purity of the eluted protein was evaluated by SDS-PAGE. The protein was then dialyzed in a buffer containing 20 mM Tris–HCl, 50 mM NaCl, and 10% glycerol (pH 8.0) and concentrated using centrifugal filters (Amicon^®^ Ultra). Protein concentration was determined using a Bradford Protein Assay Kit (Sangon Biotech) according to the manufacturer’s instructions. A standard curve was generated using bovine serum albumin (BSA) provided in the kit, and the absorbance was measured at 595 nm.

### Electrophoretic mobility shift assays

The upstream regions of genes or operons were amplified and 5′-labeled with biotin to generate probes. A total of 50–100 fmol probes were incubated with His₆-tagged CrsR protein in a binding buffer containing poly(dI-dC) for 20 min at room temperature. A 100-fold molar excess of either an unlabeled specific probe (identical to the labeled probe) or unlabeled non-specific competitor poly(dI-dC) was used in binding assays to assess the specificity of protein–probe interactions. To validate concentration-dependent binding, the assays were conducted using two concentrations of CrsR protein (0.5 and 1 μg). Protein–DNA complexes were then resolved on 8% non-denaturing polyacrylamide gels, and the band patterns were transferred to nylon membranes and UV cross-linked. Following blocking, membranes were incubated with HRP-conjugated streptavidin (Beyotime) in blocking buffer. After two washes, biotinylated probes were detected using the ECL Western Blotting Detection System (Thermo Fisher Scientific).

### Bioinformatic analysis

Sequence and conserved domain analysis of CrsR and CrsK were performed using BLAST.^1^ The three-dimensional structures of CrsR and CrsK were predicted by AlphaFold 3, with the putative binding sites of CrsR also being analyzed by the same platform^2^ (Abramson et al., 2024). The transmembrane helices of CrsK were predicted using TMHMM-2.0^3^ (Krogh et al., 2001; Sonnhammer et al., 1998).

## Results

### Bioinformatic identification of the CrsRK TCS (encoded by *CNX_RS21345/CNX_RS21350*) in the *Actinosynnema pretiosum* X47 genome

Genomic sequencing and comparative analysis revealed the presence of dozens of TCSs in the genome of the *A. pretiosum* X47 strain, most of which lack functional characterization. To systematically identify TCSs influencing AP-3 biosynthesis, we generated knockout mutants targeting the response regulator genes of multiple TCSs. The mutation in *CNX_RS21345* led to a pronounced reduction in the AP-3 titer, prompting its identification as a key candidate.

**Figure 1:**
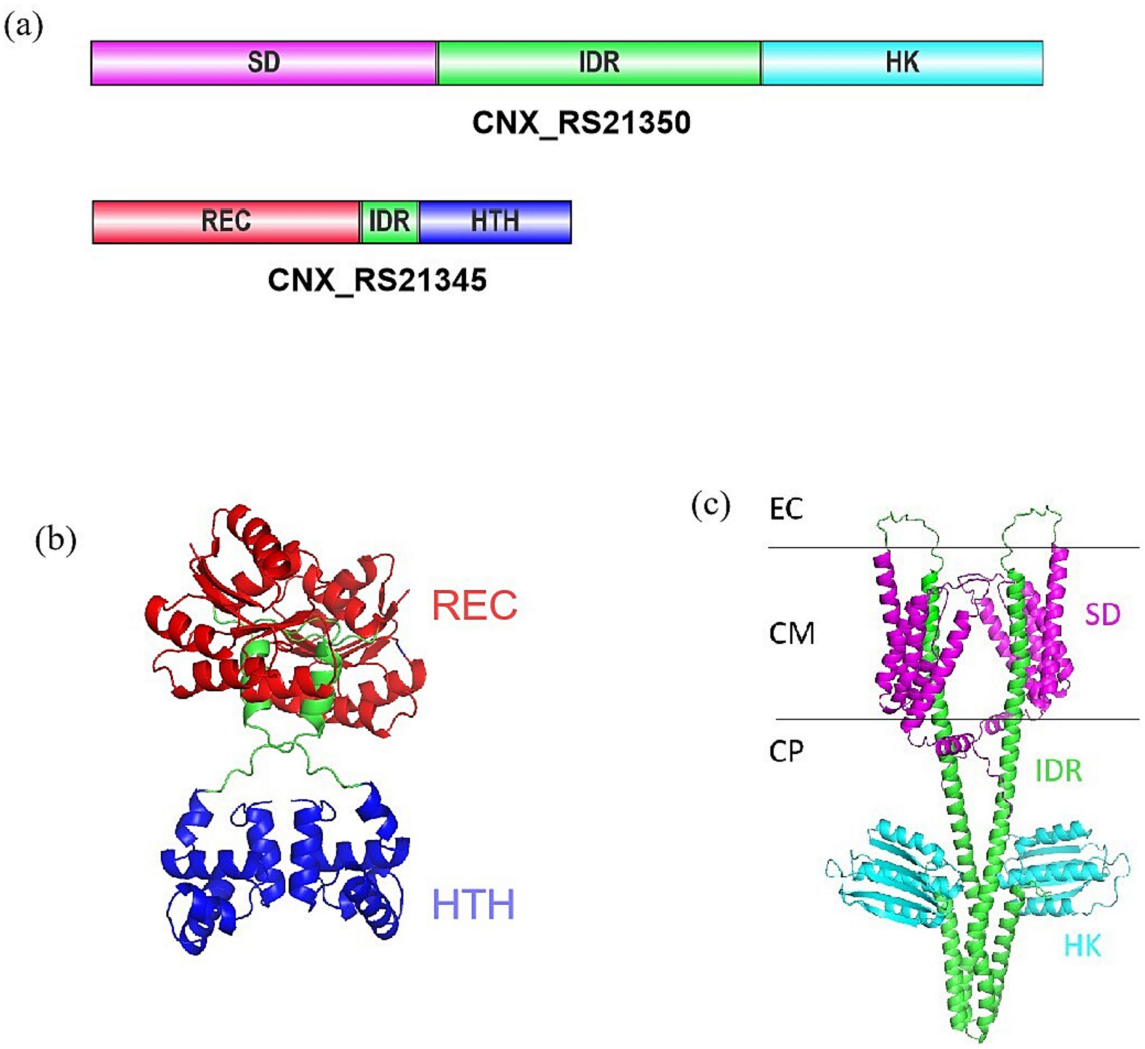
Protein structure prediction of CNX_RS21345 and CNX_RS21350. **(a)** Domain architecture of CNX_RS21345 and CNX_RS21350. **(b)** Computationally predicted the tertiary structure of CNX_RS21345. **(c)** Computationally predicted tertiary structure of CNX_RS21350. EC, extracellular; CM, cell membrane; CP, cytoplasm. Schematic representations depict conserved domains: SD, sensor domain; IDR, intrinsically disordered region; HK, histidine kinase; REC, phosphoacceptor receiver; HTH, helix-turn-helix motif.

Sequence alignment analysis identified the adjacent genes *CNX_RS21345* and *CNX_RS21350* in the X47 genome, predicted to form an operon based on their 24-bp intergenic spacer. Bioinformatic analysis identified CNX_RS21345 and CNX_RS21350 as a TCS pair. CNX_RS21345 encodes a 217-amino acid protein belonging to the NarL family of response regulators, which comprises a phosphoacceptor receiver (REC) domain and a helix-turn-helix (HTH) DNA-binding domain (Figures 1a,b). CNX_RS21350 encodes a 429-amino acid sensor histidine kinase, featuring a C-terminal histidine kinase (HK) domain and an N-terminal sensor domain (Figures 1a,c). In this study, we designated CNX_RS21345 and CNX_RS21350 as CrsR and CrsK, respectively.

### *crsR* deletion led to a significant decrease in AP-3 biosynthesis in *Actinosynnema pretiosum* X47

**Figure 2:**
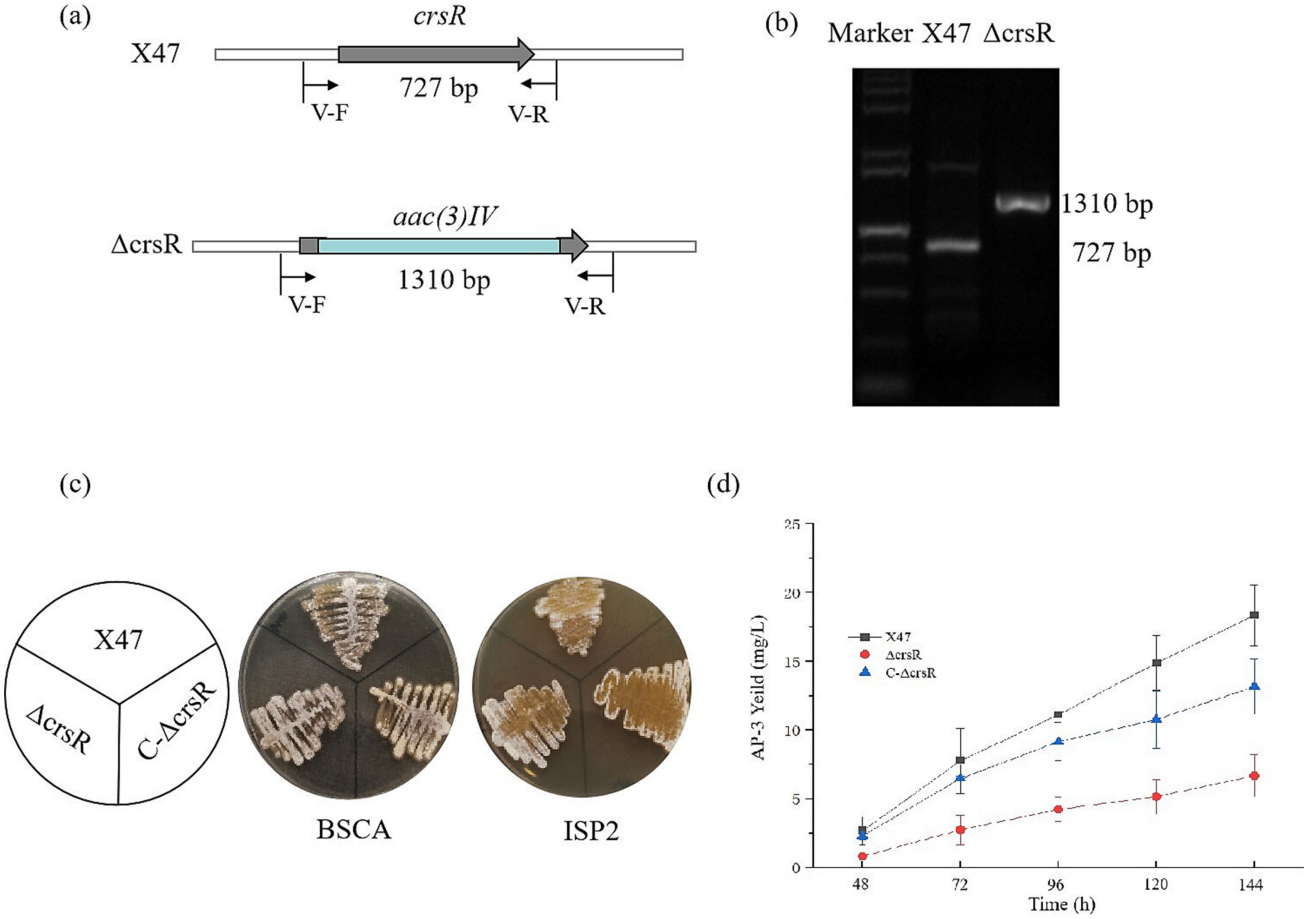
Deletion of *crsR* leads to reduced AP-3 production in *A. pretiosum* X47. **(a)** Schematic of *crsR* internal deletion (468 bp) replaced by an apramycin resistance cassette. **(b)** PCR confirmation of the *crsR* deletion using primers flanking the deletion site. **(c)** Phenotypes of *A. pretiosum* X47, ∆crsR, and C-∆crsR strains grown on BSCA and ISP2 media for 72 h. **(d)** Comparative AP-3 production in *A. pretiosum* X47, ∆crsR, and C-∆crsR strains. The results represent the mean ± SD of three independent biological replicates.

To investigate the function of CrsR, we constructed a deletion mutant of the gene *crsR* in the X47 strain. The mutant strain was verified by PCR using primer V-F/R, which produced a 727 bp amplicon in the X47 strain and a 1,310 bp fragment in the mutant strain (Figures 2a,b). Phenotypic comparison of *A. pretiosum* X47, ∆crsR, and C-∆crsR grown on BSCA and ISP2 media revealed no significant morphological differences between the ∆crsR mutant and the wild-type strain (Figure 2c). The AP-3 production in both the X47 and ΔcrsR strains was analyzed. The mutant exhibited markedly lower production levels than X47 as early as 48 h (Figure 2d). At 48 h, the X47 strain produced 2.67 ± 0.87 mg/L of AP-3, while the ΔcrsR mutant yielded only 0.82 ± 0.14 mg/L. By 144 h, AP-3 titers in X47 reached 18.36 ± 2.21 mg/L, whereas the ΔcrsR strain showed a 64% reduction (6.67 ± 1.54 mg/L) relative to the wild-type strain (Supplementary Table S2). The complemented strain showed restored AP-3 production, reaching yields similar to those of the X47 strain. Under fermentation conditions, the biomass of all strains remained similar across different time points (Supplementary Figure S1), demonstrating that the deletion of the *crsR* gene does not impact growth, which indicates that the significant differences in AP-3 production were not caused by variations in biomass.

To assess the effect of CrsR overexpression, we integrated a native *crsRS* cassette into the X47 genome, creating the X47:pMS82-*crsRS* strain, with an empty vector integrant as a control. The overexpressed strain exhibited approximately 60% enhancement in AP-3 production at 144 h relative to the control strains. Furthermore, no significant differences in biomass were observed among the three strains at either 48 h or 144 h (Supplementary Figure S2). Collectively, these results demonstrate that CrsR acts as a crucial positive regulator of AP-3 biosynthesis in *A. pretiosum*.

### *crsR* deletion causes genome-wide transcriptional changes

**Figure 3:**
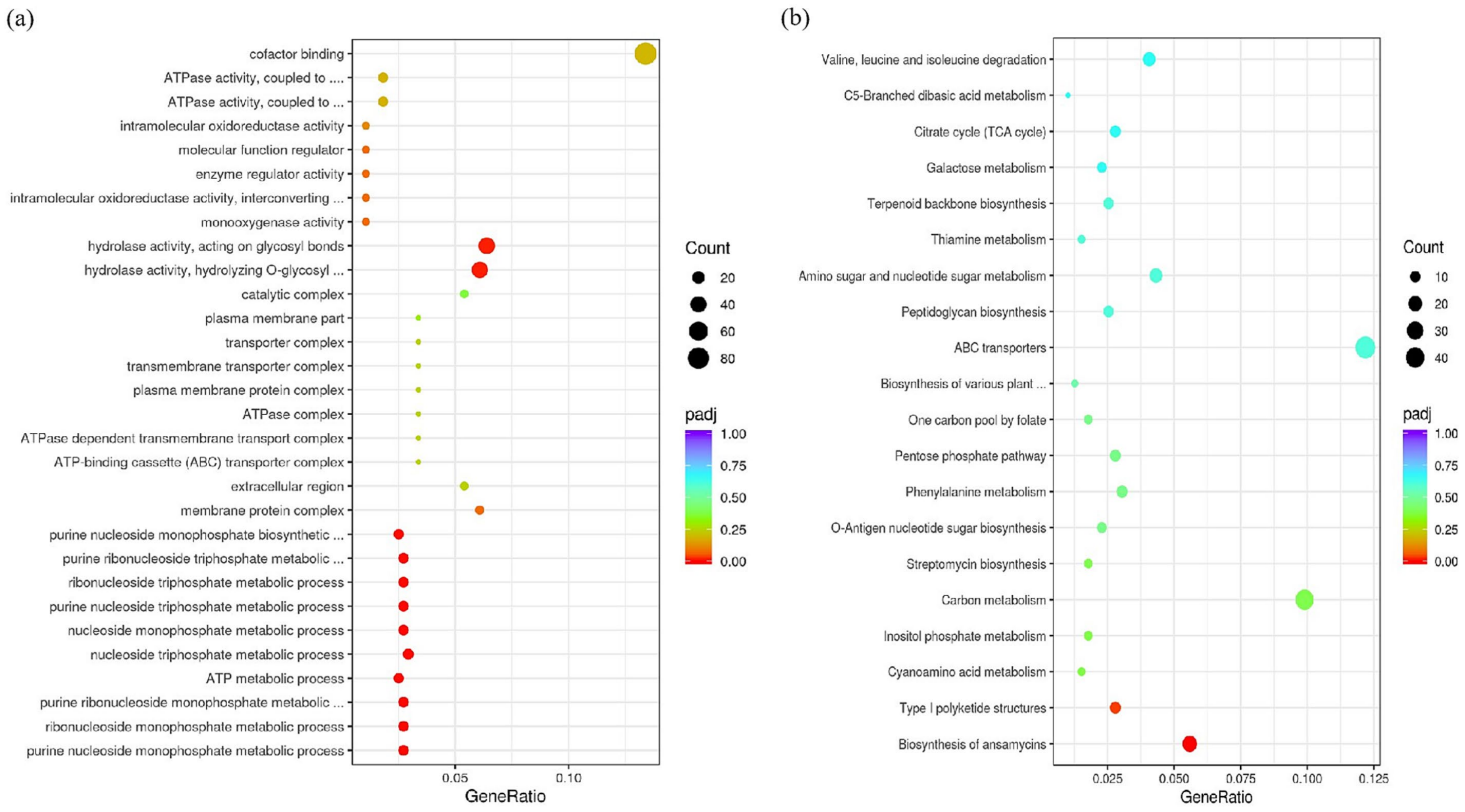
GO and KEGG enrichment analysis of DEGs in the *crsR* mutant. **(a)** Dot plot shows significantly enriched GO terms. **(b)** Dot plot shows significantly enriched KEGG pathways. The x-axis represents the gene ratio. The y-axis represents the enriched GO terms **(a)** or KEGG pathways **(b)**. Dot size corresponds to the gene count. Dot color indicates the statistical significance.

To determine how CrsR regulates AP-3 synthesis, we analyzed gene transcription in the wild-type X47 strain and the ΔcrsR mutant under fermentation conditions. The results showed that *crsR* deletion caused altered expression levels of numerous genes. At 48 h of fermentation, compared to the wild-type strain, 684 genes were upregulated and 948 genes were downregulated in the ΔcrsR mutant. GO enrichment analysis revealed that the differentially expressed genes (DEGs) were significantly associated with pathways related to purine nucleotide metabolism/biosynthesis, carbohydrate metabolic process, and nutrient catabolism (Figure 3a), suggesting that CrsR is a global regulator involved in core metabolic homeostasis.

**Figure 4:**
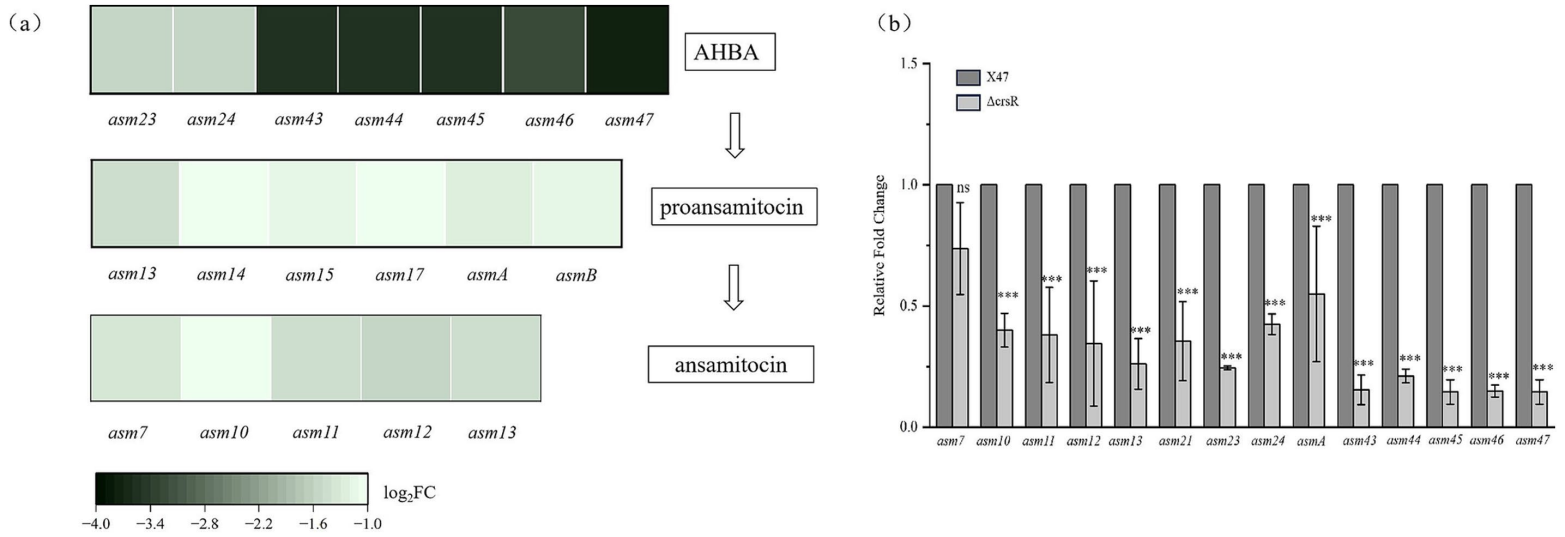
CrsR regulates the expression of AP-3 biosynthetic genes. **(a)** Heatmap depicts the significantly altered genes (*p* < 0.05) involved in the biosynthesis of AP-3. The analysis encompasses genes for AHBA biosynthesis, PKS assembly, post-assembly modifications, and tailoring steps. Each column represents a gene, and each row represents the ΔcrsR strain. The color gradient corresponds to the Log_2_ fold change (Log_2_ FC) in gene expression of the ΔcrsR strain relative to the X47 strain, with negative values indicating downregulation in the ΔcrsR mutant. **(b)** qRT-PCR validation of DEGs from the RNA-seq analysis. Expression levels were normalized to *hrdB*, and data are presented as mean ± SD from three biological replicates. Statistical significance is denoted as: ***, *p* ≤ 0.001; **, *p* ≤ 0.01; *, and *p* ≤ 0.05; ns, not significant.

Kyoto Encyclopedia of Genes and Genomes (KEGG) analysis identified ansamitocin biosynthesis as the most significantly enriched pathway (Figure 3b), consistent with altered AP-3 production in the ΔcrsR mutant. The transcriptional levels of genes involved in the AP-3 biosynthesis pathway were quantitatively assessed in the ΔcrsR mutant relative to the wild-type strain (Supplementary Table S3). Uridine Diphosphate Glucose (UDPG) serves as the starting material and is converted to the starter unit AHBA (Yu et al., 2002). The genes encoding key enzymes involved in this process, *asm23–24* and *asm43–47*, were significantly downregulated in the ΔcrsR mutant. In the proansamitocin biosynthetic pathway of the mutant strain, the transcriptional levels of the polyketide synthase (PKS) genes (*asmA, asmB*) and the post-PKS modification genes (*asm13–15*, *asm17*) were significantly downregulated (Figure 4a).

To validate the transcriptome sequencing results, we quantified the expression of *asm* genes (*asm7, asm10–13, asmA, asm23–asm24, and asm43–asm47*) in strains X47 and ΔcrsR using qRT-PCR. The results were consistent with the RNA-seq data, except for *asm7*, which showed no significant difference in expression between the ΔcrsR and X47 strains (Figure 4b). These findings collectively suggest that CrsR acts as a global regulatory factor that positively modulates the transcription of AP-3 biosynthetic genes, thereby influencing AP-3 production.

### CrsR binds to the promoters of *asm21*, *asm43–44*, and *asm45–47* genes

**Figure 5:**
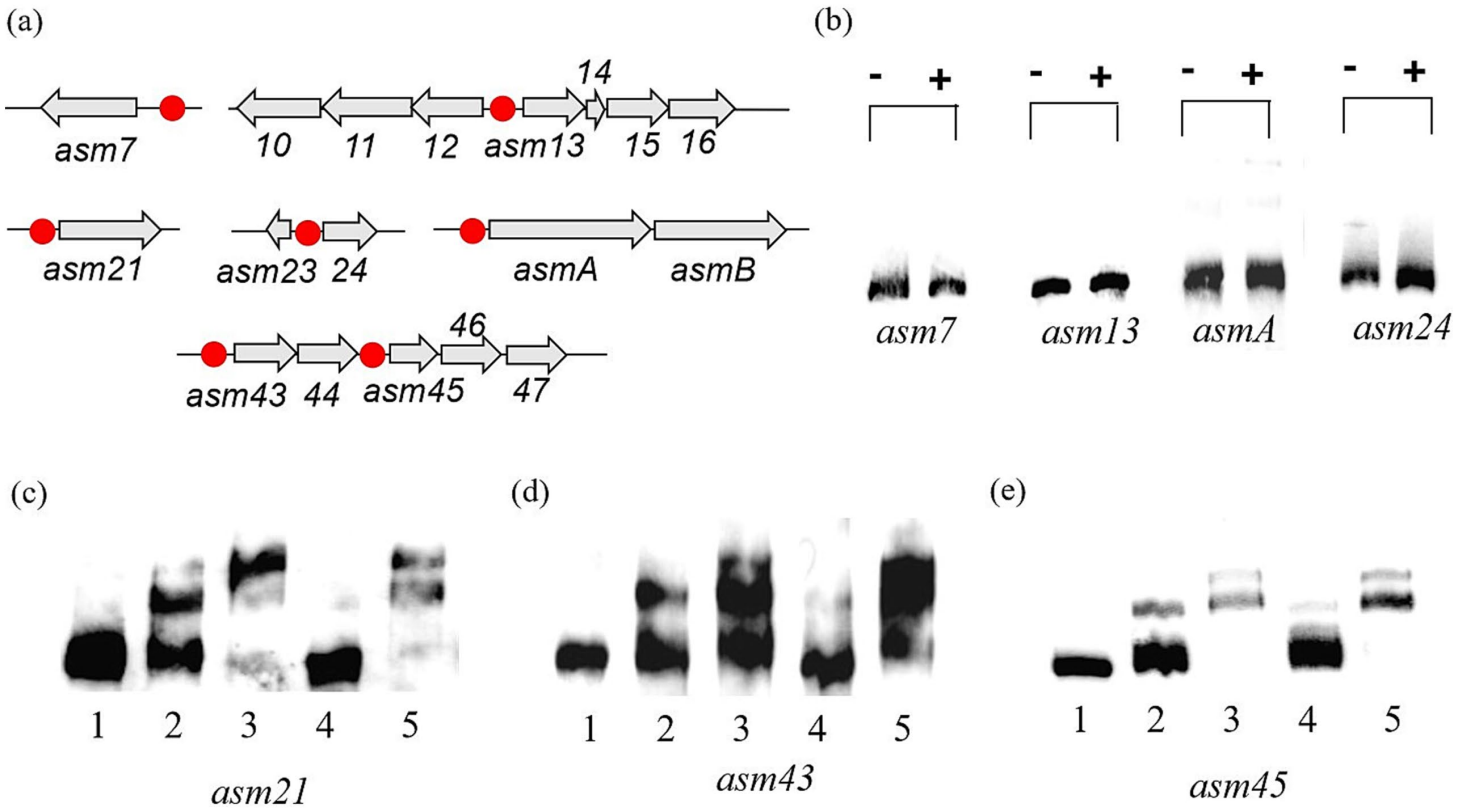
EMSAs with the His₆-tagged CrsR with target probes. **(a)** Transcriptional organization of CrsR-affected genes or operons within the *asm* cluster. Probe locations are marked with red dots. **(b)** Binding of CrsR to the *asm7*, *asm13*, *asm24*, and *asmA* promoters. (−): no protein; (+): 1 μg CrsR. **(b–d)** Binding of CrsR to the *asm21* **(c)**, *asm43* **(d)**, and *asm45* **(e)** promoters. Lane 1: probe only (no protein); Lane 2: probe + 0.5 μg CrsR; Lane 3: probe + 1 μg CrsR; Lane 4: probe + 1 μg CrsR + 100-fold molar excess of unlabeled specific probe; and Lane 5: probe + 1 μg CrsR + 100-fold molar excess of unlabeled non-specific probe.

EMSAs were performed to assess whether CrsR directly binds to AP-3 biosynthetic gene promoters. Based on operon organization determined from gene structure and RNA-seq analysis, promoter regions of *asm7*, *asm13–15* (*asm10–12*), *asm21*, *asm24, asm43–44*, *asm45–47*, and *asmA-B* were amplified and biotin-labeled as probes (Figure 5a). Our results demonstrate that the His₆-tagged CrsR protein does not bind to the promoters of *asm7*, *asm13–15* (*asm10–12*), *asm24,* or *asmA* (Figure 5b). Instead, it specifically binds to the promoters of *asm21*, *asm43–44*, and *asm45–47* (Figures 5c–e). The concentration dependence of CrsR binding was evaluated by comparing its binding activity at 0.5 μg and 1.0 μg of protein (Figures 5c–e). Compared to the control lane with no CrsR, the addition of 0.5 μg CrsR resulted in a clearly visible shifted band, indicating that binding occurred, while a significant amount of free probe remained unbound. When the CrsR concentration was increased to 1.0 μg, the signal of the shifted band intensified significantly. The results revealed a marked increase in the DNA–protein complex signal at the higher CrsR concentration, confirming that binding is concentration-dependent. In competitive binding assays, a 100-fold excess of unlabeled specific DNA fragment significantly reduced binding to the *asm21* promoter, whereas an equivalent excess of the non-specific competitor poly(dI-dC) had no effect (Figure 5c). Similar binding patterns were observed for the *asm43* and *asm45* promoters (Figures 5d,e). These findings indicate that His₆-tagged CrsR specifically interacts with the *asm21*, *asm43–44*, and *asm45–47* promoters, suggesting that it directly regulates their transcription.

We used AlphaFold 3 to predict the precise binding sites of CrsR in the promoter regions of its target genes (Supplementary Table S5). The resulting models exhibited low confidence scores, with ipTM <0.6 and pTM < 0.5. Furthermore, no clearly conserved binding motif was identified among the predicted sequences.

## Discussion

**Figure 6:**
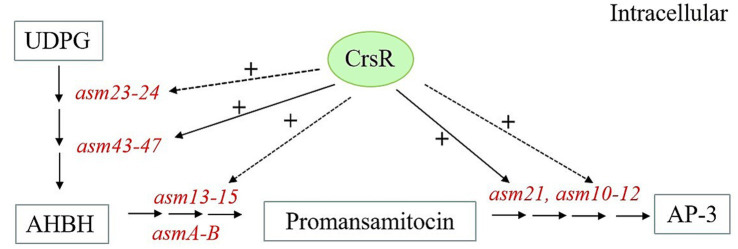
Proposed model of CrsR regulation in *asm* BGC. CrsR positively regulates AP-3 biosynthetic genes. (+) Denotes positive regulation. Direct and indirect regulations are represented by solid and dashed lines, respectively.

The *A. pretiosum* X47 genome encodes dozens of putative TCSs, most of which are uncharacterized. Notably, we functionally characterized CrsR, the response regulator of TCS CrsRK, and proposed a CrsR-dependent regulatory model for AP-3 biosynthesis (Figure 6). KEGG pathway analysis indicated that the CrsR mutation primarily disrupted the AP-3 biosynthetic pathway. The transcriptional levels of the majority of genes (*asm23–asm24* and *asm43–asm47*) in the cluster were downregulated to varying degrees, with the most pronounced changes observed for *asm43–asm47* (Yu et al., 2002). EMSA results demonstrated that CrsR directly binds to the promoters of *asm43–asm47*, which regulates the transcription of these genes, ultimately affecting the metabolic flux from UDP-glucose toward AHBA synthesis. Furthermore, reduced transcription of genes (*asm10–asm15*, *asm21,* and *asmA–asmB*) involved in the PKS pathway and post-modification steps further impaired the production of AP-3 (Spiteller et al., 2003). CrsR directly regulates *asm21*, as evidenced by its binding to the *asm21* promoter region *in vitro*. In contrast, CrsR does not directly regulate other genes, including *asm10–15, asmA*, *asmB*, and *asm23–24*. EMSAs confirmed the absence of CrsR binding to their promoter regions, indicating an indirect regulatory mechanism. Although the pathway-specific regulators within the *asm* BGC were not controlled by CrsR (Supplementary Table S4), we proposed that CrsR modulates the expression of *asm10–15*, *asmA*, and *asm23–24* through other pleiotropic regulators. However, the precise mechanism remains to be elucidated. Collectively, our results demonstrate that the response regulator CrsR acts as a global transcriptional activator of AP-3 biosynthesis, directly or indirectly regulating the *asm* BGC.

In a typical TCS, the kinase senses environmental signals, undergoes autophosphorylation, and subsequently phosphorylates its cognate response regulator (Zschiedrich et al., 2016). CrsK is the histidine kinase component of the CrsRK TCSs, equipped with a signal perception domain and a histidine kinase domain. Its proposed role is to sense external signals, autophosphorylate, and then phosphorylate the response regulator CrsR to activate downstream gene regulation. Although we speculate that the kinase domain mediates the interaction with CrsR, the exact environmental signals that CrsK detects cannot be inferred due to a lack of functional data on homologous proteins. A critical next step to fully decipher the regulatory logic of this pathway will be to systematically investigate the signals perceived by CrsK and the precise mechanism of its interaction with CrsR.

*Actinomycetes* are the predominant reservoir of bioactive natural products, accounting for most of the clinically used antibiotics (Barka et al., 2016). Antibiotic biosynthetic genes are typically organized in clusters within the genome. Antibiotic biosynthesis is governed by a hierarchical regulatory framework comprising pathway-specific control mediated by cluster-situated regulators (CSRs) and global regulation orchestrated by pleiotropic regulators, which collectively constitute an integrated network that dynamically coordinates antibiotic production (Wei et al., 2018). Knowledge of antibiotic biosynthesis regulation is crucial for discovering novel antibiotics and enhancing the yields of known compounds. For instance, in *S. coelicolor*, ACT biosynthesis is governed by the CSR ActII-ORF4 while simultaneously being regulated by global regulators including AdpA, MtrA, MacR, WblA, and DraR (Kang et al., 2007; Lee et al., 2013; Liu et al., 2021; Som et al., 2017b; Yu et al., 2012). Few research has elucidated the CSRs governing AP-3 biosynthesis in *A. pretiosum,* offering diverse strategies for yield improvement. Within the *asm* BGCs, the LuxR family regulator Asm8 directly activates the AHBA formation, while others, such as Asm18, not only increase ansamitocin production but also enhance the chemical diversity of the metabolites produced (Li et al., 2016; Pan et al., 2013). Asm2 and Asm39 function as positive regulators of ansamitocin biosynthesis, and constitutive overexpression of these genes significantly enhances AP-3 production (Ng et al., 2009). Additionally, global regulators governing AP-3 biosynthesis have been characterized. In *A. pretiosum subsp. auranticum* ATCC 31565, the AdpA family regulator AdpA_1075 pleiotropically links morphological differentiation to ansamitocin biosynthesis (Guo et al., 2022). AdpA_1075 positively regulates ansamitocin biosynthesis by directly controlling *asm28* expression. CrsR indirectly regulates the expression of genes such as *asmAB* and *asm10–15* but has no effect on the transcription of regulatory proteins within the gene cluster (Supplementary Table S4), suggesting that other regulatory pathways control the expression of the ansamitocin gene cluster. In *A. pretiosum* X47, knockout of the response regulator in the CNX_RS34865/CNX_RS34870 TCS led to reduced AP-3 biosynthesis, accompanied by significant downregulation of several *asm* genes, including *asm1*, *asm2*, *asm30*, *asm32–asm35*, and *asm37* (Zhang P. et al., 2022). Our study reveals that CrsR, the response regulator of the CrsRK TCS, acts as another key regulator in the AP-3 biosynthetic network. Notably, these two regulatory systems appear to operate independently, as RNA-seq analysis revealed that CrsR does not affect the expression of *CNX_RS34865/CNX_RS34870*, and each system regulates a distinct subset of *asm* genes. This clear segregation of target genes suggests that AP-3 synthesis is finely tuned through parallel signaling pathways, allowing the integration of different environmental or physiological cues for precise metabolic control.

Our RNA-seq data support that CrsR functions as a global regulator, affecting not only AP-3 biosynthesis but also primary metabolic processes such as purine synthesis. Although the deletion of *crsR* led to transcriptional upregulation of these metabolic genes (e.g., *guaA*, *atpA*, *atpD*, *atpF*, *atpG*, and *atpH*), AP-3 production decreased, suggesting that the enhanced metabolic flux may have been redirected toward other cellular processes rather than being channeled into AP-3 biosynthesis.

As a response regulator, CrsR is expected to specifically bind to conserved DNA motifs within the promoters of its target genes. Our data confirm that CrsR does bind directly to the promoters of *asm21*, *asm43*, and *asm45*. However, AlphaFold-based predictions failed to reveal a consistently conserved binding motif among these regions, and the resulting models exhibited low confidence scores. This apparent discrepancy may reflect inherent limitations of current structure prediction tools in accurately modeling protein–DNA interactions, particularly when binding involves flexible regions or non-canonical interfaces (Abramson et al., 2024). Therefore, in future studies, combining ChIP-seq with EMSAs and bioinformatic analyses will be essential to define the conserved binding motif of CrsR and comprehensively elucidate its regulatory mechanism.

In conclusion, this study identifies and functionally characterizes the novel TCS CrsRK in *A. pretiosum* X47, revealing its global regulatory role in AP-3 biosynthesis. We demonstrate that CrsR directly targets key biosynthetic genes (*asm21* and *asm43–47*) through promoter binding, as confirmed by EMSAs. However, the mechanistic basis of CrsR-mediated regulation, including its conserved DNA-binding motifs, potential co-regulatory partners, and the environmental signals sensed by its cognate kinase CrsK, requires further investigation. Elucidating these mechanisms will advance the fundamental understanding of how TCSs orchestrate complex secondary metabolism in *actinomycetes* and enable rational engineering of high-yield AP-3 strains.

## Data Availability

RNA-Seq data has been deposited in the Sequence Read Archive422 (SRA) under the accession number PRJNA1301260.

## Footnotes

^1^ https://blast.ncbi.nlm.nih.gov/Blast.cgi

^2^ https://alphafoldserver.com/

^3^ https://services.healthtech.dtu.dk/services/TMHMM-2.0/
