# Supplementary material for: The response regulator CrsR positively regulates ansamitocin P-3 biosynthesis in Actinosynnema pretiosum
Source: Front Microbiol. 2025 Nov 4;16:1684526. doi: 10.3389/fmicb.2025.1684526 (PMC12623338; doi:10.3389/fmicb.2025.1684526)
Supplement: Supplementary file 1 [file Data_Sheet_1.docx]

Table S1 Primers used in this study

| **Primers** | **Description or sequence (5'-3')** |
| --- | --- |
| **For gene knock-out** |  |
| crsR-L-F | ACCCGGGGATCCTCTAGAGATTTCTAGATCAGGTGCGGGCTGTCCA |
| crsR-L-R | CCAAGTTGACCAGTGCCGTTCAGCACGGTCAAGTCGCAC |
| crsR-R-F | TTTCCCTTGTCCAGATAGCCAGGTCGTCCTCGCTGTCG |
| crsR-R-R | GCATGCCTGCAGGTCGACGATAAAGCTTTGGTTCGCCGTGGAGTTCA |
| crsR-V-F | GAACCGCCGCAGAAGTGG |
| crsR-V-R | GTGATCTCCGTGGTGCTCGT |
| **For gene complement** | |
| crsR-Com-F | AAGCTTGTGACGGGCCTGATGCAGCT |
| crsR-Com-R | AAGCTTGAACCGCCGCAGAAGTGG |
| **For heterologous protein expression** | |
| crsR Ex-F | TGCCGCGCGGCAGCCATATGGTGATCTCCGTGGTGCTCGT |
| crsR Ex-R | TTAGCAGCCGGATCCTCGAGTCAGCGCGCGGCGAGGCCGTT |
| **For EMSA** |  |
| *asm7* E-F | GCCTCCTGGAACGGGTGC |
| *asm7* E-R | CGGTCCACCAGCCGCATC |
| *asm13* E-F | ACTCGGTCGCCGTGGTCA |
| *asm13* E-R | CGCACCTCCTCCAGGCTCA |
| *asmA* E-F | TGACGGCGTCGGGTGAGG |
| *asmA* E-R | GTCGCTTCGCAGCATCTCT |
| *asm24* E-F | GGTCGGGCACCAGGAACA |
| *asm24* E-R | AGCTTCTGCGGGGCCTTC |
| *asm21* E-F | GTGGATGAGCAGCATGTACTTC |
| *asm21* E-R | GGAGGGACGAGCGGAGAC |
| *asm43* E-F | CCGGTTCCCGTTCATCCA |
| *asm43* E-R | TGCCCATGAGCCCCTGTC |
| *asm45* E-F | CGGACAGACCGATCCCCGAGTC |
| *asm45* E-R | CCCGTCGAGGTCGAACAGCAC |
| **For qRT-PCR** |  |
| *hrdB* qRT-F | CAACCTCCGCCTGGTGGTGT |
| *hrdB* qRT-R | GGAGAACTTGTAGCCCTTGG |
| *asm7* qRT-F | CTCACCCGCCTGCTCACG |
| *asm7* qRT-R | GAGGAGGTCGAACAGCGG |
| *asm10* qRT-F | ACGGTGTTCTTCGACGCG |
| *asm10* qRT-R | TCCACCTCGAACACCACCAC |
| *asm11* qRT-F | GTGGAGTGGCTGTCGTCGTT |
| *asm11* qRT-R | TTCCACGCCAGGTTCTCC |
| *asm12* qRT-F | CGTCGGGCTGCTGGAGGA |
| *asm12* qRT-R | GGAACACCGCCACCATGC |
| *asm13* qRT-F | GTCATGGGCTGCGGGATCA |
| *asm13* qRT-R | GGGATGGAGGAGGTGTTGGA |
| *asm 21* qRT-F | CGGTGGCGTTCTACTTCC |
| *asm21* qRT-R | AGTGCCCGAAGGAGGACA |
| *asm23* qRT-F | TCGGGACCATCCAGGACA |
| *asm23* qRT-R | ATCCACGGGCTCGGGTA |
| *asm24* qRT-F | CGCAGAAGCTGGAGTTCCC |
| *asm24* qRT-R | CAGGACGAGTTCGAGGGCGT |
| *asmA* qRT-F | GCTGCGAAGCGACCTGAT |
| *asmA* qRT-R | GGTTGCCCAGCAGGATCA |
| *asm43* qRT-F | CCCGCCTTCACGTTCATCTC |
| *asm43* qRT-R | GTCCAGGCAGTAGGTGTCGG |
| *asm44* qRT-F | GTGGCTGCCCCGGTTGCT |
| *asm44* qRT-R | GCACAGCGGCTTCTCCAGGAA |
| *asm45* qRT-F | GTCAACAGCTTCGCCGTCAT |
| *asm45* qRT-R | CCAGGTGCCGGTTGTACTC |
| *asm46* qRT-F | CTGTGGCTGGGCACGACCGA |
| *asm46* qRT-R | AGTTCGCTGGAGGCCGTCGC |
| *asm47* qRT-F | AACCTCGTCGGCTCCTACTG |
| *asm47* qRT-R | ATGAAGTGGCAGCGGGCG |
|  |  |

Table S2 Quantification of AP-3 production in *A. pretiosum* strains

| **Strains** | **Time point**  **(h)** | **Replicate 1 (mg/L)** | | **Replicate 2 (mg/L)** | **Replicate 3 (mg/L)** | **Mean ± SD (mg/L)** |
| --- | --- | --- | --- | --- | --- | --- |
| X47 | 48 | | 3.67 | 2.24 | 2.09 | 2.67 ± 0.87 |
| ΔcrsR | 48 | | 0.97 | 0.79 | 0.69 | 0.82 ± 0.14 |
| C-∆crsR | 48 | | 1.97 | 2.64 | 2.33 | 2.31 ±0.34 |
| X47 | 144 | | 20.89 | 16.85 | 17.33 | 18.36 ± 2.21 |
| ΔcrsR | 144 | | 8.25 | 5.17 | 6.54 | 6.67 ± 1.54 |
| C-∆crsR | 144 | | 15.11 | 13.02 | 11.36 | 13.16 ±1.88 |

| **Gene ID** | **Gene annotation** | **log_2_FoldChange (ΔcrsR/X47)** | ***p* value** | **padj** |
| --- | --- | --- | --- | --- |
| CNX65_RS16255 | *asm23* | -1.580051976 | <0.01 | <0.01 |
| CNX65_RS16260 | *asm24* | -1.580422373 | <0.01 | <0.01 |
| CNX65_RS16515 | *asm43* | -3.538224458 | <0.01 | <0.01 |
| CNX65_RS16520 | *asm44* | -3.517369011 | <0.01 | <0.01 |
| CNX65_RS16525 | *asm45* | -3.597433442 | <0.01 | <0.01 |
| CNX65_RS16530 | *asm46* | -3.284046272 | <0.01 | <0.01 |
| CNX65_RS16535 | *asm47* | -3.724097753 | <0.01 | <0.01 |
| CNX65_RS16200 | *asm13* | -1.424838496 | <0.01 | <0.01 |
| CNX65_RS16205 | *asm14* | -1.055216446 | <0.05 | >0.05 |
| CNX65_RS16210 | *asm15* | -1.105699268 | <0.01 | >0.05 |
| CNX65_RS16220 | *asm17* | -1.023891304 | <0.05 | >0.05 |
| CNX65_RS37060 | *asmA* | -1.208183562 | <0.01 | <0.05 |
| CNX65_RS37065 | *asmB* | -1.171008524 | <0.01 | <0.05 |
| CNX65_RS16135 | *asm7* | -1.364303354 | <0.01 | <0.05 |
| CNX65_RS16185 | *asm10* | -1.014715819 | <0.05 | >0.05 |
| CNX65_RS16190 | *asm11* | -1.473885886 | <0.01 | <0.01 |
| CNX65_RS16195 | *asm12* | -1.520566612 | <0.01 | <0.01 |
| CNX65_RS16240 | *asm21* | -1.463200091 | <0.01 | <0.01 |

Table S3 Downregulation of AP-3 biosynthesis genes in ΔcrsR strain compared to the X47 strain (p<0.05 and/or padj<0.05)

Table S4 Comparative analysis of cluster-situated regulator (CSR) gene expression within the *asm* BGC between the X47 and ΔcrsR strains

| **Gene ID** | **Gene annotation** | **RNA-seq^a^**  **(ΔcrsR/X47)** | **qRT-PCR^b^**  **(ΔcrsR/X47)** | ***p* value** |
| --- | --- | --- | --- | --- |
| CNX65_RS16110 | *asm2* | \| 1.081947053 \| \| --- \| | 1.18±0.27 | >0.05 |
| CNX65_RS16140 | *asm8* | - | 1.14±0.20 | >0.05 |
| CNX65_RS16225 | *asm18* | \| 1.165273494 \| \| --- \| | 1.45±0.64 | >0.05 |
| CNX65_RS16345 | *asm34* | \| 1.555127535 \| \| --- \| | 1.44±0.45 | >0.05 |
| CNX65_RS16495 | *asm39* | 1.195143457 | 1.53±0.24 | >0.05 |

a：represents the fold change (ΔcrsR/X47) from RNA-seq data.

b：represents the fold change (ΔcrsR/X47) from qRT-PCR (mean ± SD, n=3).

Table S5 Prediction of CrsR binding sites on target gene promoters using AlphaFold 3

| **Target gene** | **Putative DNA binding sites for CrsR** | **ipTM^a^** | **pTM^b^** |
| --- | --- | --- | --- |
| *asm21* | TGGCGCAAGGGATTA | 0.29 | 0.4 |
| *asm43* | TTCCCCGAAAGCGTCACCG | 0.26 | 0.35 |
| *asm45* | CTGCTCCGGCGACCGCCG | 0.24 | 0.34 |

1. Interface predicted template modeling (ipTM) scores below 0.6 are indicative of a low-confidence prediction.
2. Predicted template modeling (pTM) scores below 0.5 suggest a lack of global structural similarity to the true structure.


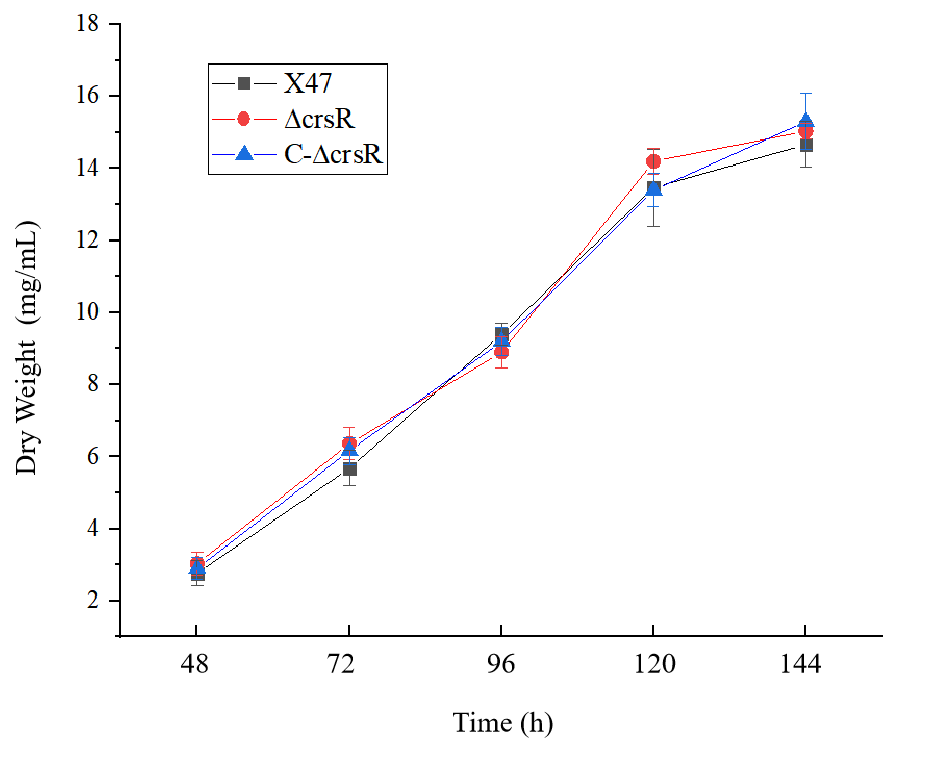


Fig. S1 Biomass of the X47, ΔcrsR, and C-ΔcrsR strains grown in fermentation medium. Dry weight represent the mean ± SD from three independent biological replicates of *A. pretiosum*strains.


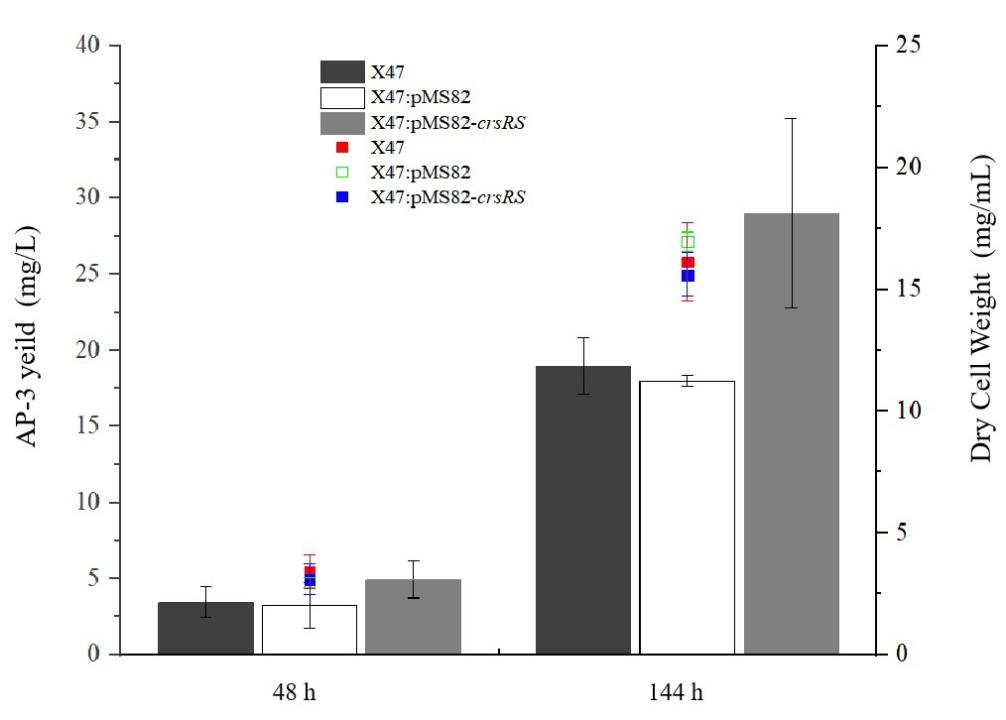


Fig. S2 Effects of CrsR overexpression on AP-3 production and cell growth in *A. pretiosum*. AP-3 yield (left Y-axis, rectangle) and dry cell weight (right Y-axis, square) in the wild-type (X47), empty vector control (X47:pMS82), and CrsR-overexpressing (X47:pMS82-*crsRS*) strains at 48 h and 144 h. Results represent the mean ± SD of three independent biological replicates.
